# Effects of Supplemental Calcium Propionate and Concentrate Level: Growth Performance, Body Fat Reserves, and Health of High-Risk Beef Calves

**DOI:** 10.3390/vetsci11080336

**Published:** 2024-07-25

**Authors:** Alejandro Rivera-Villegas, Octavio Carrillo-Muro, Daniel Rodríguez-Cordero, Pedro Hernández-Briano, Oliver Yaotzin Sánchez-Barbosa, Rosalba Lazalde-Cruz, Beatriz Isabel Castro-Pérez, Alejandro Plascencia

**Affiliations:** 1 Unidad Académica de Medicina Veterinaria y Zootecnia, Universidad Autónoma de Zacatecas, General Enrique Estrada 98500, Mexico; alejandro.rivera@uaz.edu.mx (A.R.-V.); danielroco@uaz.edu.mx (D.R.-C.); pedro.hernandez@uaz.edu.mx (P.H.-B.); oliver.sanchez@uaz.edu.mx (O.Y.S.-B.); 2 Instituto de Investigaciones en Ciencias Veterinarias, Universidad Autónoma de Baja California, Mexicali 21100, Mexico; rosalba.lazalde@uabc.edu.mx; 3 Facultad de Medicina Veterinaria y Zootecnia, Universidad Autónoma de Sinaloa, Culiacán 80260, Mexico; isabel.castro@uas.edu.mx

**Keywords:** beef calf, costs, energy diet, gluconeogenic precursors, hematological responses, income, newly received, serum metabolites

## Abstract

**Simple Summary:**

During the reception stage, received beef calves are considered “high risk”, experiencing low feed intake, which leads to negative energy balance and increased susceptibility to diseases. This can result in high morbidity and mortality and substantial economic losses. For this reason, the primary goal at receiving is to boost nutrient intake, which can be accomplished by enhancing the energy content of the diet. This can be achieved by increasing levels of concentrate (CON), along with calcium propionate (CaPr) at daily doses of 20 g/hd/d. The findings suggest a daily CaPr supplementation of 20 g and a CON level of 50% over a period of 42 d significantly enhances growth performance, dietary utilization, and economic return in high-risk calves.

**Abstract:**

The aim of this study was to examine the impact of daily calcium propionate (CaPr) supplementation (0 or 20 g/calf) on growth performance, dietary energetics, body fat reserves, serum metabolites, and hematological responses in high-risk beef calves fed diets with varying (50, 60, or 70%) concentrate (CON) levels. In addition, a cost/income analysis of CaPr supplementation was carried out. Forty-eight crossbred bull calves (152.8 ± 1.56 kg body weight and 5.5 months of age) were involved in a fully randomized experimental design employing a 2 × 3 factorial arrangement of treatments. Calves were allocated (*n* = 8 per treatment) to individual pens (3.14 × 5.25 m) and were subjected to one of the following treatments during 42 d: No CaPr supplementation in diets containing 50, 60, or 70% CON (NoCaPr + 50, NoCaPr + 60, NoCaPr + 70, respectively) or daily CaPr supplementation dosed at 20 g/calf in diets containing 50, 60, or 70% CON (20CaPr + 50, 20CaPr + 60, 20CaPr + 70, respectively). Non-supplemented calves exhibited decreased dry matter intake (DMI) with increasing CON levels in their diets, while CaPr-supplemented calves displayed the opposite effect (interaction, *p* = 0.04). In calves fed a lower-CON diet (50%), those supplemented with CaPr showed greater average daily gain (ADG, 20.2%, *p* = 0.05) and lower DMI (2.2%, *p* = 0.03), resulting in improved ADG/DMI ratio, dietary energy, and energy retention (24.6, 14.4, and 18%, *p* < 0.05). These effects diminished when calves received diets with 60 or 70% CON but led to a 14.2% increase in rump fat thickness (*p* = 0.04). Only in non-supplemented CaPr calves, increasing the level of CON from 50 to 70% in the diet increased ADG (21.2%), decreased DMI (2.2%), and improved the ADG/DMI ratio (22.7%), with no impact on dietary net energy utilization. Non-supplemented calves exhibited an increase in lymphocytes as CON levels rose in their diets, whereas CaPr-supplemented calves showed the opposite effect (interaction, *p* = 0.05). Supplementation of CaPr decreased total protein (TP, *p* = 0.03) and albumin (ALB, *p* < 0.01) serum concentrations, with lower concentrations observed in 20CaPr + 50. CaPr supplementation reduced (*p* = 0.01) total cholesterol (TCHO) levels. An interaction between CaPr and CON level (*p* = 0.02) was observed since TCHO levels remained consistently low at higher CON levels. Glucose was decreased with increasing levels of CON (*p* = 0.02) but not (*p* = 0.85) for CaPr-supplemented calves. NoCaPr + 50 and NoCaPr + 70 increased (*p* = 0.05) ALB concentration. Gamma glutamyltransferase levels increased (*p* = 0.05) with increasing CON levels irrespective of CaPr supplementation. Comparing the profit within the same CON level in the diet, CaPr treatments yielded higher income, with the largest difference in profit observed when CaPr was supplemented at 50% CON level (USD 29 more/calf). In conclusion, CaPr supplementation proves to be an effective strategy for enhancing growth performance and dietary energy among high-risk beef calves, resulting in greater economic returns. The groups that received CaPr demonstrated superior profitability, particularly in calves fed diets with lower CON levels. Under the conditions in which this experiment was carried out, the optimal response occurred when the low-CON diet (50%) was supplemented with CaPr.

## 1. Introduction

One of the main concerns in feedlots when dealing with newly received calves is the observed lower dry matter intake (DMI) during the first two weeks after being received, which typically is lower than 1.5% of their body weight (BW). The target intake level to reach is 1.5% of BW (DM basis) by d 14 and increase it to 2–2.5% at three weeks after arrival [1,2,3,4]. This persistent low DMI during the initial period not only hampers the rapid recovery from weight loss during transportation but also predisposes the calves to clinical symptoms, which can occur at a frequency of up to 60% [5]. Consequently, increasing the nutrient density in diets during this phase becomes crucial to fulfill nutritional requirements, particularly for energy, as it fosters improved growth performance and health [6,7,8,9].

Protein [10] and energy [11,12] are the most essential nutrients for newly received beef calves. A sufficient energy intake enables optimal growth expression during the early stages of life [13,14] and supports a robust immune system [15]. Therefore, increasing concentrate level (CON) in receiving diets (≥60% CON) could potentially mitigate the adverse effects of low energy intake and improve immune function [6,16].

However, it has been reported that increasing CON from 45 to 66% in receiving diets for calves results in a 17% increase in the morbidity rate and a 24% rise in the number of days requiring medical treatment during the first 42 d after being received [17]. To circumvent these potential issues, receiving diets typically incorporate a proportion of forage ranging from 40 to 50% [18]. While this approach reduces the morbidity rate by 1.3% and improves DMI by 9%, it concurrently diminishes average daily gain (ADG) by 8.3% [19,20]. The reduction in morbidity achieved through high-forage receiving diets fails to offset the economic losses associated with reduced ADG [7,12,21,22]. Furthermore, optimizing the growth rate during the initial period with high-energy diets can lead to increased weight gain, yielding both short- and long-term positive effects on productivity [23]. In this sense, several strategies have been employed to the increase caloric density of receiving diets while mitigating negative repercussions, such as the inclusion of supplemental fats [24,25,26] and reducing starch concentration as the primary energy source [17], among others.

Currently, there has been growing interest in gluconeogenic precursor sources as alternative energy sources distinct from starch. In this sense, the inclusion of the glucogenic precursor calcium propionate (CaPr) has been shown to enhance energy intake and growth performance in feedlot cattle [27,28]. However, results have been inconsistent. The magnitude of response to CaPr supplementation can be explained by dosage level, diet composition, and duration of supplementation. In a trial conducted by Rodriguez-Cordero et al. [9], different daily intake levels of CaPr ranging from 20 to 80 g were tested in newly received calves for a period of 56 d. The feeding program involved a gradual increase in CON levels in the diet from 50 to 70%. Based on feed efficiency and health status responses, the optimal daily dose of CaPr and duration of supplementation were determined as 20 g and 28 d of supplementation. Calves receiving supplementation of 20 g over 56 d exhibited no changes in DMI or water consumption but mainly demonstrated increased ADG. The most significant improvements in ADG and ADG/DMI ratio were observed during the initial 28 d in calves fed a low-CON diet level (50%). The enhancements in body fat reserves (12th rib fat thickness, FAT, and rump fat thickness, RFT) were observed in calves receiving a high-CON diet (70%) with 42 and 56 d of supplementation. These results suggest that daily CaPr supplementation at a rate of 20 g in arrival calves receiving a low CON level (~50%) can be an effective strategy to improve growth performance and health, comparable to calves receiving a high-CON diet upon arrival.

Hence, in this study, we hypothesized that with daily CaPr supplementation at 20 g/calf/d, a CON level of 50% can be an effective strategy to improve growth performance, dietary energetics, FAT and RFT, serum metabolite concentration and hematologic responses, and cost/income, comparable to calves receiving a diet of 60 or 70% CON. Consequently, a trial was conducted to investigate the effects of daily CaPr supplementation (0 or 20 g) on growth performance, dietary energetics, FAT and RFT, serum metabolites, and hematological responses in newly received high-risk beef calves fed diets containing different CON levels (50, 60, or 70%). In addition, a cost/income analysis of CaPr supplementation was carried out.

## 2. Materials and Methods

The experiment took place at the Torunos Livestock Preconditioning Center, within the experimental area, Fresnillo, Zacatecas, Mexico (23°08′56.22″ N and 102°43′48.57″ W), owned by Grupo Exportador Pa Lante S.P.R. de R.L. All animal care procedures adhered to acceptable practices and experimental protocols reviewed and approved by the Unidad Académica de Medicina Veterinaria y Zootecnia at the Universidad Autónoma de Zacatecas (UAMVZ-UAZ)—Institutional Bioethics and Animal Welfare Committee (Protocol # 2023/11/02).

### 2.1. Cattle Processing

The calves utilized in this experiment were classified as high risk due to being recently weaned, having received no vaccinations, not having been castrated or dehorned, being comingled, and having been moved through an auction market [29]. In addition, they presented an unknown health and management history and a low weight condition (<200 kg) [9]. Ninety-four recently weaned crossbred (Continental × British crossbreed) bull calves from different origins were purchased and transported approximately 4 h to the Torunos, arriving on 6 November 2023. Upon arrival, the received calves were provided with free-choice oat hay, water, and mineral supplements for 12 h. The following morning (0600 h), the calves underwent processing, which included (1) metaphylactic treatment with oxytetracycline (5 mg/kg BW); (2) injection with 5 mL vaccination for clostridial organisms; (3) treating for control of internal (4% ivermectin) and external parasites (pour-on cypermethrin); (4) application of a sequentially umbered identification tag in the left ear; (5) recording of individual initial BW (IBW) (electronic scale; Metrology^®^ PBG-3000, cap. 2000 kg, Básculas y Accesorios de Peso SA de CV, Nuevo Leon, Mexico); and (6) visual clinical exam, including taking body temperature. After rehydration, weight, and health management processing, 46 calves were excluded from the experiment due to their low IBW, health problems (diarrhea, bad respiratory condition, injuries), or temperament issues (i.e., extreme fear related to handling, aggression, kicking, apathetic), resulting in a final group of 48 calves selected for the study (*n* = 48, 8 calf/treatment), averaging 152.8 ± 1.56 kg in IBW and 5.5 months of age, predominantly crossbreds. The calves were individually housed in 48 pens (8 pens [calves]/treatment) with a soil surface area of 3.14 m by 5.25 m, with 1.1 m of linear bunk space and 1 bucket of 30 L capacity for water.

### 2.2. Diet Management, Feeding, and Health Management

During the 42 d experimental phase, basal diets were formulated to meet the nutrient requirements of calves as specified by NASEM [8] (Table 1). Complete basal diets were prepared weekly using a 25 m^3^ capacity horizontal mixer (model 950 Haycruncher J&A) as follows: As the first step, the grain portion and dry supplement (mineral supplement) were added to the mixer, mixing them for a minimum of 3 min; then, coarse-ground forage hay was added to the mixer, and after 2 min of mixing, vegetable fat was added as the third step and mixed for at least 1 min. Finally, as the last step, molasses cane was added to the mixer, and when all ingredients were incorporated, they were mixed for approximately 5–7 min. After mixing, the elaborated experimental diets were stored in 1000 kg capacity bags and carefully identified for later use. To prevent cross-contamination between treatments, the mixer underwent thorough cleaning between each batch.

Each calf was provided fresh feed three times daily at 0800, 1200, and 1600 h. The amount of feed given at 0800 and 1200 h remained constant, while the feed offered at 1600 h was adjusted daily, ensuring a residual feed of approximately ~100 g/calf. Residual feed was collected daily between 0730 and 0750 h each morning to determine DMI. The residual feed of each calf collected each morning was kept in sealed bags and stored at 4 °C. The composited samples through each week were analyzed for DM until the experiment was finalized. Calves were weighed just prior to the morning feeding at the start the experiment and on day 42 (final BW [FBW], concluding the trial).

The daily water intake (DWI) was determined by daily collection of the remaining water in the bucket, registered daily at 0700 h by dipping a graduated rod into the 30 L capacity bucket drinker (Fairgate Rule #FG14-101, Thomaston, CT, USA). Once the measure was taken, the remaining water was drained, and buckets were cleaned and refilled with fresh water.

The calves were monitored daily for any signs of bovine respiratory distress, including labored breathing, nasal or ocular discharge, showing depression, anorexia, lethargy or abnormal appetites. No morbidity or mortality was observed during the study.

### 2.3. Experimental Design and Treatments

**Figure 1:**
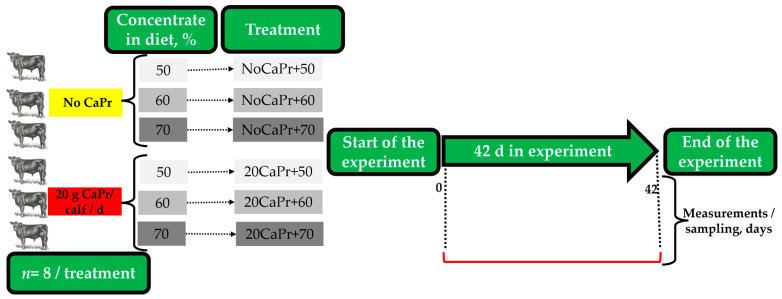
A completely randomized design experiment using 48 calves individually housed (8 calves/treatment). Treatments consisted in a daily calcium propionate (CaPr) supplementation (0 or 20 g)/calf and diets which contained different concentrate levels (CON%, 50, 60, or 70%) during a feeding period of 42 d.

The experiment followed a completely randomized design with a 2 × 3 factorial arrangement of treatments, examining the effect of daily CaPr supplementation (0 or 20 g) with diets containing three CON levels (50, 60, or 70%). The following treatments were randomly assigned to the experimental units, which were coded as (1) NoCaPr + 50; (2) NoCaPr + 60; (3) NoCaPr + 70; (4) 20CaPr + 50; (5) 20CaPr + 60; and (6) 20CaPr + 70. The experiment lasted 42 d (Figure 1). The source of CaPr used containing 20% calcium and 69% propionic acid. Each individual CaPr dose was meticulously weighed using a precision balance (Pioneer-PX523, Ohaus Corp., Parsippany, NJ, USA). CaPr treatments were offered in equal quantities in two feedings (0800 and 1600 h). To ensure CaPr consumption, the dosage per calf was mixed into 100 g of basal diet, stored in plastic bags, and provided just before the 0800 and 1600 h feedings. Any remaining portion of the diet was administered to the calves once they had consumed their initial portions.

### 2.4. Growth Performance and Dietary Energetics Calculations

Individual data collected during the feeding trial were used to calculate growth performance metrics: (1) The residual feed of each calf collected each morning was weighed, composited, and its DM content was determined through the experiment. To determine the average feed intake, the total feed offered (DM basis) minus total feed refusal (DM basis) was divided between the number of days of feeding. (2) The ADG was determined by subtracting the IBW from the FBW and dividing by the number of days on test (42) [(IBW − FBW/42] expressed as kg/d. (3) Gain efficiency (ADG/DMI ratio) was computed by dividing ADG by the daily DMI. (4) The DWI resulted from the difference between the water offered and the water refused, which was determined and recorded daily.

Dietary NE ratio and the observed-to-expected DMI ratio were calculated based on measures of growth performance (observed DMI, ADG, and average SBW) for each treatment by means of the quadratic formula according to the procedure described by Zinn et al. [31] and Lofgreen and Garrett [32].

### 2.5. Ultrasound Measurements, Serum Metabolites, and Hematolgy Analysis

Ultrasound measurements, serum metabolites, and hematology responses were determined with the procedure described by Rodriguez-Cordero et al. [9] and Russell and Roussel [33].

### 2.6. Cost/Income Analysis

A cost/income analysis was performed using growth performance (IBW, FBW, and DMI). Calculations: (1) feed cost = (DMI, kg/d × price of feed kg) × days on feed, (2) processing practice = metaphylactic antimicrobial treatment + vaccination + deworming + pour-on cypermethrin + ear tag, (3) CaPr supplementation = (CaPr, kg/d × price of CaPr kg) × days on feed, (4) cost/phase = feed + processing practice + CaPr supplementation, (5) income (selling calves) = (weight out − weight in) × price of BW/kg to calves, (6) net income = income (selling calves) − cost/phase, and (7) difference = CaPr treatments − control.

The analysis considered data for a 42 d period, calculating costs (feed, processing practice, CaPr supplementation, and cost/phase) and incomes (net income and difference). The price of feed with 50% CON (USD 347.2/ton.), 60% CON (USD 352.76/ton.), 70% CON (USD 358.31/ton.), and CaPr (USD 4.38/kg) was obtained from the feed industry (FORRVET SA de CV, Durango, Mexico). The values for the metaphylactic antimicrobial treatment (USD 1.38/calf), vaccination (USD 0.43/calf), deworming (USD 0.75/calf), pour-on cypermethrin (USD 0.63/calf), and ear tag (USD 0.61/calf) were obtained from an FORRVET S.A. de C.V. The price of calves (USD 3.62/kg BW) was obtained from the Pa Lante, enterprise for the Zacatecas region. Excel^®^ (Office 365, Microsoft, Redmond, WA, USA) was used for costs and income calculations, and results were compared between treatments using descriptive statistics.

### 2.7. Statistical Analyses

All the data were tested for normality using the Shapiro–Wilk test. Growth performance data (weight gain, gain efficiency, and dietary energetics), DMI, DWI, and blood data were analyzed as a completely randomized design with a 2 × 3 factorial arrangement of treatments, with the calf as the experimental unit, using the PROC MIXED procedures of SAS^®^ software 9.3 [34] with treatment as fixed effect and the experimental unit (calf) within treatment as a random effect. The measures of FAT, RFT, and LMA (longissimus muscle area) taken at day 0 were used as covariate to measures registered at day 42. DWI was analyzed as repeated measures. The statistical model encompassed the fixed effects of CaPr level (0 or 20 g), CON levels (50, 60, or 70%), and CaPr level × CON levels interaction. Treatment effects were considered significant when the *p*-value was ≤0.05. In addition, Tukey’s multiple comparison procedures were used. Comparisons between the differences in economic income from calves without CaPr and supplemented with CaPr were performed with the *t*-test using PROC TTEST in SAS^®^ software 9.3 [34].

## 3. Results

### 3.1. Growth Performance, Dietary Energetics, Body Fat Reserves, and Longissimus Muscle Area

There were no effects of CON level or CaPr supplementation on DWI, averaging 21.2 ± 2.2 L/d. Non-supplemented calves decreased 3% for DMI as CON levels increased beyond 60% in diets, while CaPr supplemental calves showed the opposite effect, with higher DMI (2.2%) at the low level of CON (interaction; *p* < 0.01, Table 2). However, comparing only within the group that consumed the treatment with lower CON in diet, calves receiving CaPr showed greater ADG (20.2%, *p* = 0.04) and lower DMI (2.2%, *p* = 0.03); thus, ADG/DMI ratio, dietary energy, and energy retention were improved 24.6, 14.4, and 18%, respectively (*p* < 0.05, Table 3). These positive effects vanished when calves received 60% or 70% CON, but at a high CON level, CaPr increased RFT by 14.2% (*p* = 0.04). Only in the non-supplemented calves did the CON level increasing from 50% to 70% increase ADG (21.2%), decrease DMI (2.2%), and increase ADG/DMI ratio (22.7%) without effect on the efficiency of dietary NE utilization. Finally, there were no effects of CON level or CaPr supplementation on FAT (*p* = 0.33) and LMA (*p* = 0.89), averaging 3.44 mm and 32.9 cm^2^, respectively.

### 3.2. Enzymatic Activity and Serum Metabolites

Gamma glutamyltransferase (GGT) was increased 28.6% (*p* = 0.05, Table 4) as CON levels increased independently of CaPr supplementation. Total cholesterol (TCHO) was decreased 21.7% in non-supplemented calves that were fed a 70% CON level, while for calves receiving CaPr supplementation, TCHO remained constantly low (averaging 84.4 mg/dL) at all levels of CON (interaction; *p* = 0.05, Table 5). Furthermore, CaPr affected total protein (TP, *p* = 0.03) serum concentrations, showing lower concentration (5.5 vs. 6.6 mg/dL) with 20CaPr + 50. Compared to non-supplemented calves, CaPr decreased TCHO (10.5%, *p* = 0.01). The high CON levels affected albumin (ALB) concentration, increasing it (*p* = 0.04).

### 3.3. Hematological Responses

Non-supplemented calves exhibited an increase in lymphocytes (LYM) as CON increased in diets, whereas CaPr-supplemented calves displayed the opposite effect (interaction, *p* = 0.05; Table 6). However, CaPr had no significant effects on white blood cells count. Calves that received intermediate CON levels, showed a lower granulocytes (GRA) count (*p* = 0.03) compared to calves fed lower and higher CON levels. Finally, platelets (PLT) and red blood cells (RBC) were unaffected by CaPr supplementation or CON level (*p* > 0.05, Table 7). All calves maintained counts within the normal reference intervals (RIs).

### 3.4. Cost/Income Analysis

The income analysis demonstrates that calves fed the lowest CON level with CaPr supplementation (20CaPr + 50) experienced a notable increase of USD 29.0 per calf (*p* < 0.001) during the initial 42 d of reception (Table 8). However, as the CON level increased to 60% (20CaPr + 60) and 70% (20CaPr + 70), the income gradually decreased in a linear fashion, reaching USD 8.3 (*p* < 0.01) and USD 1.2 (*p* < 0.05) per calf, respectively. This suggests a diminishing economic benefit as the CON level rises beyond the initial optimal supplementation level.

## 4. Discussion

The hypothesis raised was that newly received calves that consume a low-CON diet (50%) and are daily supplemented with 20 g of CaPr can have similar performance and health to those calves that are received with diets containing greater quantities of CON (60 or 70%). Furthermore, due to the rapid ruminal degradation and glucogenic characteristics of CaPr, it can improve the efficiency (observed-to-expected NE ratio) of dietary energy. However, no synergism was detected; on the contrary, a lower energy efficiency of CaPr in calves that were fed with high-CON diets was observed. It has been postulated that the rate of carbohydrate degradation regulates the rumen acetate:propionate ratio; at greater degradation rates, more propionate is produced [43]. However, recent findings from Lin et al. [44] indicated that the ruminal acetate:propionate ratio is directly regulated by the rumen microflora structure rather than the rate at which carbohydrates are degraded in the rumen. The explanation why the positive effects of CaPr in high-CON diets are less is uncertain and needs more research. In vitro and in vivo, CaPr feed has been shown to shift volatile fatty acid production in favor of propionate over acetate [45]. When glycerol is combined with concentrates, there may be less opportunity to improve energy efficiency than if glycerol were fed with forage because propionate production typically increases with concentrate feeding. In this sense, Drouillard [46] reported that glycerol (a glucogenic precursor) is 15% energetically less efficient when fed as part of a starch-based diet (60% CON) compared to when it is supplemented with a forage-based diet.

### 4.1. Growth Performance and Dietary Energetics

Drinking water is a critical nutrient for the growth of beef cattle on a feedlot. Any factor that limits its consumption (water quality, availability, among others) greatly affects the productivity and health of livestock [47]. Because water consumption is closely related to DMI, the importance of water in newly received cattle should cover rehydration and promote consumption during that phase [48]. Therefore, the concern about factors (such as the use of additives) that may affect water consumption is always present. In line with our results, it has been reported that the use of glucogenic precursors in receiving diets did not affect DWI in calves [9,49]. In regard to the CON level, high digestible matter requires a lower quantity of water to favor digestion and transport in the gastrointestinal tract. Therefore, lower percentages of CON in receiving diets (and thus greater forage in the diet) promoted a greater DWI [16]. In the current experiment, increasing CON in diets from 50 to 70% did not affect DWI.

Currently, there is a pursuit to increase the caloric density of diets for newly received beef calves without negative health effects through various feeding strategies such as supplemental fats [24,25,26], grain substitution as primary source of energy [17], and more recently, supplementation of the gluconeogenic precursor CaPr, which provides energy differently from starch safely [9].

In the particular case of CaPr, this is ruminally hydrolyzed into Ca^2+^ and propionic acid [50], subsequently causing improvements in rumen fermentation and ruminal bacterial diversity [51]. The main changes are related to alterations of volatile fatty acid patterns [52], increasing the proportion of propionic acid [53] with a reduction in methane production.

In addition, it been observed that CaPr increases digestibility of DM [54] and reduces the NH_3_-N ruminal concentration [55] and blood urea nitrogen (BUN) [9]. In the same manner, CaPr improves insulin response in glucose metabolism [56,57] and increases adipogenesis [58], impacting FAT and RFT depots [28]. Cumulatively, all these mechanisms promote an enhanced energy state [50]. Therefore, a better productive performance can be expected when CaPr is supplemented, mainly during the first days of receiving the calves in the feedlot. Improving the nutrient plane, mainly energy, during the receiving at the feedlot becomes crucial to the fulfill nutritional requirements of newly received calves, as it fosters improved growth performance and health [59]. In the present experiment, calves receiving CaPr, particularly those feeding with low-CON diets, showed improvements in ADG and feed efficiency similar to those non-supplemented calves that received high CON levels. Although the reports about of the effects of supplemental CaPr in newly received calves are very limited, a previous report [9], like our study, indicated improvements in DMI, ADG, and ADG/DMI ratio in newly received calves supplemented daily with 20 g of CaPr but feeding under a transition diet system. The energy optimization favored by CaPr ingestion is in line with the observed-to-expected dietary net energy ratio observed in this experiment for calves receiving CaPr at low levels of CON. Based on the observed growth performance and the expected diet net energy in accordance with tabular values for individual feed ingredients, the observed-to-expected ratio can be used to estimate the dietary energy utilization efficiency. It is a more accurate and useful method of expressing differences in energy utilization for growth performance than the conventional measure of “feed efficiency” [31]. When observed-to-expected dietary net energy ratios are set at 1.00, they indicate that observed ADG is consistent with what should be expected based on DM intake and the energy density of the diet. Improvements in energy efficiency utilization are expressed by a ratio greater than 1.00, whereas a ratio below 1.00 indicates inefficient energy usage from feed. Therefore, the energy utilization of feed was improved by 14% in calves receiving CaPr at low levels of CON. The lower impact of CaPr in calves that were fed with high-CON diets could be explained such that the benefits on fermentation mediated by CaPr are minimized by the effects that the high intake of starch had on ruminal fermentation.

In the current experiment, the increased CON levels increased ADG by 21% and slightly reduced DMI by 2%, improving feed efficiency, which is consistent with what is reported previously [6,14,16,22,24,60]. However, in some experiments, differences in ADG or DMI between diets containing different energy concentrations were not observed [17,24,61]. The discrepancies between the studies may be due to differences in the conditions of the received calves such as breed, nutritional background, health status, weight loss (shrink) during transportation and handling, the study design, the energy levels tested, or the feeding program used during the first 42–56 d. For example, in experiments where the compared energy levels are very low (i.e., <1.9 Mcal net energy for maintenance, NE_m_), no differences in ADG are detected [17,24]. This may be explained because early findings [16] suggest that differences in improved performance can be detected with diets containing greater energy densities than 1.95 Mcal net energy for gain (NE_g_)/kg. In the case of Tomsczak’s study, the feed management strategy varied between treatments such that the DMI of the diets which had greater energy density was limited to a greater extent than the lower-energy diets relative to ad libitum DMI. This could avoid the differences in daily energy intake between treatments. Either way, a meta-analysis conducted by Rivera et al. [7] also concluded, just like our results, with non-supplemented calves, which increased dietary energy densities and improved DMI and ADG in newly received calves.

### 4.2. Body Fat Reserves and Longissimus Muscle Area

Body fat reserves indicate the nutritional status of cattle [62]. Energy or protein intake over requirements of protein deposition stimulates the deposition of body fat reserves [63,64]. In the present experiment, with the exception of calves that received CON 50 diets without receiving CaPr vs. receiving CaPr (7.41 vs. 7.15 Mcal NE_m_/d, respectively), due to differences in DMI between non-supplemented vs. supplemented calves, the daily energy intake was quite similar for calves receiving CON at the 60 and 70% levels, being 8.00 vs. 7.91 and 8.30 vs. 8.40 Mcal NE_m_ for non-supplemented vs. supplemented calves, respectively. In such a manner, the difference in fat deposition in the body and LMA can be explained mainly by the energy contribution of supplemental CaPr. According to Mendoza-Martínez et al. [65], the energy contribution of CaPr is similar to propionic acid, and gross energy of 3.965 Mcal/kg is equivalent to ME of 3.766 Mcal/kg. The NE_m_ of CaPr can be estimated at 2.54 Mcal NE_m_/kg by the partial energy efficiency of ruminants to convert EM to NE_m_ [66]; this energy is 7% over the energy supply of the steam-flaked corn grain (2.38 Mcal NE_m_/kg) assigned by NASEM [8]. Considering that the energy level is the main factor that affects the rate of weight in cattle [31,67] and in a parallel manner the LMA [68], the effects observed in CaPr-supplemented calves are consistent with those expected. Our results are in line with those reported previously [9,14,69] in which the LMA, FAT, and RFT of newly received calves are increased as a consequence of CaPr supplementation.

### 4.3. Enzymatic Activity and Serum Metabolites

All blood enzymes and metabolites measured were within normal RIs across the treatments [35]. The assessment of ALP (alkaline phosphatase), GGT, AST (aspartate aminotransferase), and ALT (alanine aminotransferase) enzyme activity aimed to determine whether different CON levels and CaPr supplementation could modify hepatic or renal functions or improve metabolism in these organs. The increase in GGT activity in both high CON levels or CaPr supplementation can be attributed to increases in hepatic activity [70]. In a previous study, a tendency for increased GGT in newly received calves when supplemented daily with 20 g CaPr was observed [9].

Regarding ALP activity, no effects were observed within the RI (RI = 0 to 488; [35]), which was also noted with CON levels [71]. However, high doses of CaPr (80 g/calf/d) have been reported to cause reductions [9], which, although remaining within the RI, could be associated with a reduction in osteoblasts (bone growth reduction) in young growing cattle [40]. The RI for AST (RI = 48 to 100; [36]) was unaffected by CON or CaPr levels, indicating an nonspecific indicator of no tissue injury [72]. These results were similar to those obtained with increasing doses of CaPr [9], CON [71], or glycerin [73,74], although other authors did report increases when glycerin was increased [75]. Likewise, no changes were observed in the RI for ALT (RI = 11 to 40; [35]), consistent with other authors working with increased CON levels [14]. Ultimately, the values of ALP, GGT, AST, and ALT activity were below the pathological RIs, suggesting no hepatic or renal damage or improvements in the metabolism of these organs associated with CaPr supplementation or different CON levels in newly received beef calves.

TP is the main solid component of serum, consisting of ALB and globulins (GLO), and is an important indicator of the nutritional status of cattle [76]. In the present study, CaPr reduced TP concentrations (RI = 6.7 to 7.5; [35]) and ALB (RI = 2.7 to 4.2; [36]), being lower with low CON levels (20CaPr + 50), which coincides with Li et al. [77], who observed that with low levels of CON, TP decreased but no changes were observed in ALB, but these values remained within the normal RIs. However, with different CaPr [9], CON [71], or glycerin [74] levels, no effects were observed. Regarding GLO, no treatment effects were observed; however, elevated CaPr levels raised it [9] (RI = 3.0 to 3.48; [35]); likewise, increases in GLO have been reported with the increase in CON [77]. The increase in concentrations of ALB, GLO, or both is known as hyperproteinemia, and it can be solely caused by animal dehydration. However, if there is hyperproteinemia but the calves are not dehydrated, it is almost always a result of hyperglobulinemia, commonly caused by antigen stimulation (chronic inflammatory disease, such as chronic pneumonia) or liver diseases [33]. Based on the aforementioned principles, we can assume that the increasing use of CON may stimulate chronic inflammation, and CaPr supplementation with low levels of CON reduces it.

BUN levels are used to estimate N excretion and its utilization efficiency [78]. In the current study, N utilization efficiency was not affected by treatments (RI = 10 to 25; [37]); other authors observed similar effects with different CON levels [14,22]. However, BUN levels decrease with low CaPr levels [9]. Nevertheless, with high doses of CaPr [27], CON [77], or crude glycerin [49,74], N utilization efficiency decreases, as observed in calves with immunological problems, where muscular catabolism increases to obtain more proteins and improve immune response [79]. Protein catabolism is higher (elevated BUN) at the beginning of the receiving phase (first 20 d), later decreasing because the animal relies more on dietary protein than muscular catabolism [22].

Creatinine (CRE) concentrations were not affected by different CON levels and CaPr supplementation, being within the normal RI (RI = 1 to 2; [35]), indicating adequate renal glomerular filtration rate and no interference from treatments; likewise, it has been reported that increasing CON [14,71] or glycerin [72,74] has no effect. However, it has also been observed that CRE levels increase with increasing CaPr inclusion [9].

Total bilirubin (TBIL) is an important indicator of liver function as it increases during severe lipidosis [80,81] and decreases when the liver is healthy. Reductions in TBIL have been reported with the increase in CON [77]. However, in the current study, TBIL concentrations were not affected by different CON levels and CaPr supplementation, being within the normal RI (RI = 0.01 to 0.5; [35]) and indicating that these treatments have no negative effects on liver function.

Lipids primarily consist of TCHO and triglycerides (TG), reflecting liver energy metabolism, particularly lipid export in the form of very-low-density lipoproteins [82]. Diets high in CON, and all supplemented with CaPr, showed the lowest TCHO levels but remained within the RI (RI = 73 to 280; [37]); however, high doses of CaPr [9] or crude glycerin [74,75] increase concentration, probably due to increased propionic acid production in the rumen, consequently increasing TCHO production in the liver, with decreased levels indicating energy deficit [83]. No treatment effects were observed for TG; however, all values were above the RI (RI = 0 to 14; [35]), consistent with Rodriguez-Cordero et al. [9] and Carrillo-Muro et al. [27].

Serum calcium (Ca, RI = 8.3 to 10.4; [37]) levels were not affected by different CON levels or CaPr supplementation. However, as CaPr supplementation levels increase, Ca concentration rises [9,84].

Plasma glycemic levels are considered metabolic indicators of nutrient intake in beef cattle [85]. The different treatments maintained glucose (GLU) within the normal RI (RI = 45 to 75; [35]); nevertheless, low CON levels without CaPr supplementation further elevated GLU, consistent with glycerin reduction [72] or CON levels [77]. But, various authors note that supplementation with different CaPr levels [9,27,53,57], CON [14,22], or crude glycerin [74,75] has no effect on glycemia; nonetheless, Ermita et al. [86] observed increases with CaPr administration. GLU concentrations from all treatments were above the normal RI, related to adequate DMI, as glycemic levels are influenced by nutrient availability [87]. Concentrations above the normal RI suggest energy improvement associated with efficient dietary nutrient utilization [88].

The results of this study indicate that different CON levels or CaPr supplementation have no effect on the normal RIs of sodium (Na^+^, RI = 132 to 152; [35]), potassium (K^+^, RI = 3.9 to 5.8; [35]), and chlorine (Cl^−^, RI = 97 to 97–111; [35]). Similarly, the inclusion of different CaPr levels [9] or CON [14] had no effect on electrolyte concentrations. All of the above indicates that basal diets perfectly met the electrolyte nutritional requirements of the calves. In spite of that, low Na^+^ and Cl^−^ values result from diets not fully meeting nutritional requirements [89] or due to diarrhea [72], and K^+^ deficiency is commonly associated with stressed cattle, resulting from dehydration and K^+^ loss in tissues [48].

### 4.4. Hematological Responses

Different CON levels and CaPr supplementation had no effect on white blood cells, and normal RIs of total white blood cells (WBCs, RI = 4 to 12; [38]), LYM% (RI = 45 to 75; [39]), monocytes (MONs, RI = 0 to 0.8; [40]), MON% (RI = 45 to 75; [40]), and GRA% (RI = 15 to 45; [41]); likewise, increasing CaPr levels did not show changes in these variables [9]. Maintaining LYM within the normal RI (RI = 1.6 to 5.6; [39]) indicates a healthy immune status of the calves. However, it was observed that as CON increased without CaPr supplementation, LYM also increased beyond the RI. Conversely, with increasing CON supplemented with CaPr, the opposite occurred; LYM decreased to within the normal RI. Similarly, increasing CaPr inclusion doses reduced LYM in newly received beef calves [9], or with crude glycerin inclusion [74,75,90]. Regarding GRA, this increased with the highest and lowest CON levels; the same happened with high CaPr levels [9], but all remained within the normal RI (RI = 1.8 to 6.3; [41]).

Different CON levels and CaPr supplementation had no effect and did not modify the normal RIs of PLT (RI = 193 to 637; [41]), mean platelet volume (MPV, RI = 4.5 to 7.5; [41]), RBCs (RI = 5.1 to 7.6; [41]), red blood cell distribution width test % (RDW%, RI = 16 to 20; [41]), hemoglobin (HGB, RI = 58.0 to 12.0; [41]), hematocrit (HCT, RI = 22 to 32; [41]), mean corpuscular volume (MCV, RI = 38 to 50; [39]), mean corpuscular hemoglobin (MCH, RI = 14–19; [42]), and mean corpuscular hemoglobin concentration (MCHC, RI = 38 to 43; [42]). Similarly, no effects were observed with different CaPr levels [9] or with crude glycerin inclusion [74].

### 4.5. Costs and Income Analysis

Because CaPr improved ADG and feed efficiency independent of the level of CON in the diet, beef calves supplemented with CaPr showed overall more income/calf than non-upplemented calves. These improvements with CaPr supplementation were more pronounced as the CON level in the diet was lower; consequently, income per calf/42 d increased as the CON level decreased (USD 1.3, USD 8.3, and USD 29.0/calf, for 70, 60, and 50% CON, respectively).

## 5. Conclusions

Supplementing 20 g CaPr/calf with 50% CON for 42 d improves growth performance, dietary energetics, and increases incomes. This improvement is reflected in increased ADG, ADG:DMI ratio, and energy utilization and retention, all leading to increased economic incomes. Likewise, increasing CON plus CaPr inclusion (60 or 70 + CaPr) increases RFT and reduces LYM. However, different levels of CaPr and CON did not affect the RI of most serum metabolites and had no negative effect on the hematological responses of beef calves.

## Figures and Tables

**Table 1:** Ingredients of the basal diets offered to calves and chemical composition (g kg^−1^ DM).

Ingredients	Concentrate in Basal Diets, %		
	50	60	70
Alfalfa hay, mature	250.0	200.0	150.0
Oat hay	250.0	200.0	150.0
Corn grain, cracked	280.0	380.0	480.0
Soybean meal-44	105.0	105.0	105.0
Molasses cane	50.0	50.0	50.0
Soybean oil	21.5	21.5	21.5
Bentonite, sodium	7.5	7.5	7.5
Sodium sesquicarbonate	15.0	15.0	15.0
Calcium carbonate	8.0	8.0	8.0
Monocalcium phosphate	2.0	2.0	2.0
Urea	5.0	5.0	5.0
Salt	5.0	5.0	5.0
Microminerals/vitamins	1.0	1.0	1.0
Chemical composition, g/kg DM			
Dry matter	872.40	862.42	852.41
Crude protein	148.83	146.52	144.10
Ether extract	43.64	45.84	48.01
Neutral detergent fiber	348.42	298.71	249.02
Starch ^a^	23.00	29.44	35.90
Calcium ^a^	9.31	8.95	8.54
Phosphorus ^a^	2.92	2.84	2.81
Calcium:Phosphorus ratio	3.20	3.21	3.01
Calculated net energy, Mcal/kg			
Maintenance	1.57	1.69	1.81
Gain	0.98	1.08	1.18

^a^ Calculated based on the tabular values for individual feed ingredients (Starch, Ca, P, NE_m_ and NE_g_) [8], with the exception of DM, CP (Leco^®^ FP-428, nitrogen analyzer), NDF (Ankom 220 fiber analyzer), and EE (extractor of Ankom^xt15^), which were determined in our laboratory [30].

**Table 2:** Effects of supplemental calcium propionate (CaPr) and concentrate level (CON) on growth performance in high-risk beef calves.

Item ^a^	Calcium Propionate, g/calf/d ^b^						SEM ^c^	Significance		
	0			20						
	Concentrate in Diet, % ^b^							CON	CaPr	CON × CaPr
	50	60	70	50	60	70				
Daily water intake, L/d	21.1	21.0	21.5	20.9	20.5	22.1	0.91	0.53	0.93	0.85
Initial body weight, kg	148.53	148.12	148.18	142.31	147.01	144.61	3.65	0.85	0.27	0.80
Final body weight, kg	190.77 ^b^	194.75 ^ab^	198.75 ^a^	192.92 ^ab^	196.72 ^ab^	196.73 ^ab^	4.37	0.39	0.04	0.69
Average daily gain, kg/d	0.998 ^b^	1.110 ^ab^	1.210 ^a^	1.202 ^a^	1.181 ^ab^	1.239 ^a^	0.07	0.46	0.04	0.51
Dry matter intake, kg/d	4.719 ^ab^	4.737 ^a^	4.588 ^c^	4.560 ^c^	4.686 ^ab^	4.642 ^bc^	0.03	<0.01	0.03	<0.01
ADG/DMI ratio	0.211 ^b^	0.234 ^ab^	0.259 ^ab^	0.263 ^a^	0.252 ^ab^	0.261 ^ab^	0.01	0.4	0.05	0.32

^a^ ADG = average daily gain, DMI = dry matter intake. ^b^ Treatments consisted of oral administration of CaPr (0 or 20 g/calf/d), combined with 3 concentrate levels in diet (50, 60, or 70%) for period of 42 d. ^c^ SEM = standard error of the mean. ^a–c^ Within a row, means without a common letter superscript differ (*p* < 0.05).

**Table 3:** Effects of supplemental calcium propionate (CaPr) and concentrate level (CON) on dietary energetics and ultrasound measurement in high-risk beef calves.

Item	Calcium Propionate, g/calf/d ^a^						SEM ^b^	Significance		
	0			20						
	Concentrate in Diet, % ^a^							CON	CaPr	CON × CaPr
	50	60	70	50	60	70				
Dietary NE (Mcal/kg)										
Maintenance	1.629 ^b^	1.739 ^ab^	1.868 ^a^	1.858 ^ab^	1.814 ^ab^	1.850 ^ab^	0.06	0.34	0.05	0.29
Gain	1.019 ^b^	1.102 ^ab^	1.228 ^a^	1.202 ^ab^	1.181 ^ab^	1.212 ^ab^	0.06	0.34	0.05	0.29
Observed: expected dietary NE ratio										
Maintenance	1.033 ^b^	1.029 ^b^	1.032 ^b^	1.182 ^a^	1.073 ^ab^	1.022 ^b^	0.04	0.07	0.04	0.21
Gain	1.039 ^b^	1.020 ^b^	1.016 ^b^	1.243 ^a^	1.092 ^ab^	1.019 ^b^	0.05	0.08	0.04	0.17
Ultrasound measurements										
12th rib fat thickness, mm	3.23	3.41	3.54	3.44	3.46	3.57	0.15	0.24	0.33	0.73
Rump fat thickness, mm	3.84 ^b^	3.86 ^b^	4.18 ^b^	4.08 ^b^	4.50 ^a^	4.66 ^a^	0.29	0.33	0.04	0.82
Longissimus muscle area, cm^2^	32.38	32.44	34.14	32.13	33.32	33.06	1.31	0.66	0.89	0.81

^a^ Treatments consisted of oral administration of CaPr (0 or 20 g/calf/d), combined with 3 concentrate levels in diet (50, 60, or 70%), for period of 42 d. ^b^ SEM = standard error of the mean. ^a,b^ Within a row, means without a common letter superscript differ (*p* < 0.05).

**Table 4:** Effects of supplemental calcium propionate (CaPr) and concentrate level (CON) on enzymes activity in high-risk beef calves.

Item	Calcium Propionate, g/calf/d ^a^						Reference Intervals	SEM ^b^	Significance		
	0			20							
	Concentrate in Diet, % ^a^								CON	CaPr	CON × CaPr
	50	60	70	50	60	70					
Alkaline phosphatase, U/I	201.1	266.7	200.5	177.3	198.5	204.6	0–488 [35]	28.87	0.31	0.21	0.47
Gamma glutamyltransferase, U/I	14.9	17.9	17.1	12.1	20.0	20.6	6.1–17.4 [35]	2.52	0.05	0.74	0.41
Aspartate aminotransferase, U/I	61.2	73.2	66.4	72.9	72.7	73.6	48–100 [36]	5.16	0.51	0.15	0.53
Alanine aminotransferase, U/I	23.5	22.9	24.2	25.1	24.8	26.0	7–35 [36]	1.12	0.52	0.06	0.98

^a^ Treatments consisted of oral administration of CaPr (0 or 20 g/calf/d), combined with 3 concentrate levels in diet (50, 60, or 70%) for period of 42 d. ^b^ SEM = standard error of the mean.

**Table 5:** Effects of supplemental calcium propionate (CaPr) and concentrate level (CON) on serum metabolites in high-risk beef calves.

Item	Calcium Propionate, g/calf/d ^a^						Reference Intervals	SEM ^b^	Significance		
	0			20							
	Concentrate in Diet, % ^a^								CON	CaPr	CON × CaPr
	50	60	70	50	60	70					
Total protein, g/dL	6.7 ^ab^	6.5 ^ab^	6.8 ^a^	5.9 ^b^	6.5 ^ab^	6.6 ^ab^	6.74–7.46 [35]	0.21	0.12	0.03	0.15
Albumin, g/dL	3.4 ^ab^	3.2 ^b^	3.6 ^a^	3.2 ^b^	3.2 ^b^	3.2 ^b^	2.8–3.8 [36]	0.07	0.04	<0.01	0.11
Globulins, g/dL	3.3	3.3	3.3	3.0	3.3	3.4	3.0–3.48 [35]	0.11	0.21	0.78	0.25
Blood urea nitrogen, mg/dL	11.5	12.1	11.5	12.8	11.6	10.9	10–25 [37]	0.58	0.26	0.87	0.21
Creatinine, mg/dL	0.9	0.8	1.0	0.9	1.5	0.8	1–2 [35]	0.21	0.46	0.31	0.12
Total bilirubin, mg/dL	0.5	0.4	0.6	0.8	0.6	0.6	0.01–0.5 [35]	0.23	0.88	0.33	0.77
Total cholesterol, mg/dL	102.3 ^a^	102.4 ^a^	80.1 ^b^	85.7 ^ab^	81.6 ^ab^	86.1 ^ab^	80–120 [37]	5.12	0.81	0.01	0.02
Triglycerides, mg/dL	18.3	21.6	20.8	19.1	17.8	18.5	0–14 [35]	2.37	0.87	0.39	0.65
Calcium, mg/dL	11.0	10.4	10.6	10.4	10.8	10.5	8.3–10.4 [37]	0.21	0.71	0.63	0.09
Glucose, mg/dL	93.7 ^a^	86.6 ^ab^	76.3 ^b^	89.8 ^ab^	84.0 ^ab^	85.3 ^ab^	45–75 [35]	4.01	0.02	0.85	0.18
Electrolytes, mEq/L											
Sodium	137.5	137.4	136.1	119.8	137.6	137.4	132–152 [35]	3.22	0.33	0.33	0.34
Potassium	5.4	6.0	6.0	5.6	5.6	5.9	3.9–5.8 [35]	0.23	0.14	0.58	0.31
Chlorine	97.1	99.4	97.3	97.8	97.7	99.0	97–111 [35]	0.77	0.31	0.65	0.11

^a^ Treatments consisted of oral administration of CaPr (0 or 20 g/calf/d) combined with 3 concentrate levels in diet (50, 60, or 70%), for period of 42 d. ^b^ SEM = standard error of the mean. ^a,b^ Within a row, means without a common letter superscript differ (*p* < 0.05).

**Table 6:** Effects of supplemental calcium propionate (CaPr) and concentrate level (CON) on white blood cells in high-risk beef calves.

Item	Calcium Propionate, g/calf/d ^a^						Reference Intervals	SEM ^b^	Significance		
	0			20							
	Concentrate in Diet, % ^a^								CON	CaPr	CON × CaPr
	50	60	70	50	60	70					
Total white blood cells, ×10^3^/μL	11.8	10.2	12.7	11.2	11.0	10.5	4–12 [38]	0.71	0.28	0.24	0.11
Lymphocytes, ×10^3^/μL	6.4	6.2	7.2	6.9	6.3	5.8	1.6–5.6 [39]	0.42	0.63	0.39	0.05
Lymphocytes, %	55.1	61.0	59.3	60.9	58.5	56.0	45–75 [39]	4.33	0.54	0.99	0.06
Monocytes, ×10^3^/μL	1.0	0.9	1.5	1.4	0.9	1.4	0–0.8 [40]	0.33	0.27	0.72	0.80
Monocytes, %	8.0	8.3	8.0	7.6	7.8	8.0	2–7 [40]	0.26	0.54	0.14	0.55
Granulocytes, ×10^3^/μL	4.4	3.0	4.5	3.4	3.3	3.8	1.8–6.3 [41]	0.38	0.03	0.19	0.24
Granulocytes, %	36.9	30.6	33.7	31.5	33.7	36.0	15–45 [41]	1.95	0.35	0.99	0.06

^a^ Treatments consisted of oral administration of CaPr (0 or 20 g/calf/d) combined with 3 concentrate level in diet (50, 60, or 70%), at period of 42 d. ^b^ SEM = standard error of the mean.

**Table 7:** Effects of supplemental calcium propionate (CaPr) and concentrate level (CON) on platelets and red blood cells in high-risk beef calves.

Item ^a^	Calcium Propionate, g/calf/d ^b^						Reference Intervals	SEM ^c^	Significance		
	0			20							
	Concentrate in Diet, % ^b^								CON	CaPr	CON × CaPr
	50	60	70	50	60	70					
Platelets, ×10^3^/µL	230.3	253.5	251.9	256.3	295.7	236.0	193–637 [41]	21.94	0.31	0.34	0.42
Mean platelet volume, fL	7.3	6.9	7.8	7.1	7.0	7.0	4.5–7.5 [41]	0.25	0.24	0.16	0.22
Red blood cells, ×10^6^/μL	9.9	9.9	10.2	10.1	9.8	9.3	5.1–7.6 [41]	0.32	0.71	0.32	0.27
RDW, %	27.5	26.3	25.8	26.3	27.4	26.4	16–20 [41]	0.53	0.26	0.73	0.07
Hemoglobin, g/100 mL	12.4	12.7	12.7	12.4	12.3	12.0	8.0–12.0 [41]	0.32	0.90	0.21	0.57
Hematocrit, %	33.6	35.2	34.4	34.6	33.7	33.1	22–32 [41]	1.10	0.81	0.49	0.44
Red blood cell index											
MCV, fL	34.1	35.7	34.2	34.6	34.5	35.8	38–50 [39]	0.66	0.41	0.55	0.11
MCH, pg	12.5	12.9	12.8	12.4	12.6	13.1	14–19 [42]	0.29	0.22	0.88	0.62
MCHC, g/dL	36.8	36.1	37.9	35.9	36.6	36.6	38–43 [42]	0.68	0.34	0.28	0.37

^a^ RDW% = red blood cells distribution width test %, MCV = mean corpuscular volume, MCH = mean corpuscular hemoglobin, MCHC = mean corpuscular hemoglobin concentration. ^b^ Treatments consisted of oral administration of CaPr (0 or 20 g/calf/d) combined with 3 concentrate levels in diet (50, 60, or 70%) for period of 42 d. ^c^ SEM = standard error of the mean.

**Table 8:** Effects of supplemental calcium propionate (CaPr) and concentrate level (CON) on cost/income estimated for high-risk beef calves during the experimental phase (42 d).

Item	Calcium Propionate, g/calf/d ^a^					
	0			20		
	Concentrate in Diet, % ^a^					
	50	60	70	50	60	70
Costs, USD/calf						
Feed ^b^	USD 68.80	USD 70.20	USD 69.00	USD 66.50	USD 69.50	USD 69.80
Processing practice ^c^	USD 3.80	USD 3.80	USD 3.80	USD 3.80	USD 3.80	USD 3.80
CaPr supplementation ^d^	USD 0.00	USD 0.00	USD 0.00	USD 3.70	USD 3.70	USD 3.70
Cost/phase	USD 72.60	USD 74.10	USD 72.80	USD 74.00	USD 77.00	USD 77.30
Income, USD/calf						
Income (selling calves) ^e^	USD 153.00	USD 168.80	USD 183.10	USD 183.30	USD 180.00	USD 188.70
Net income	USD 80.30	USD 94.80	USD 110.30	USD 109.30	USD 103.00	USD 111.40
Difference	-	-	-	USD 29.00 ***	USD 8.30 **	USD 1.20 *

^a^ Treatments consisted of oral administration of CaPr (0 or 20 g/calf/d), combined with 3 concentrate levels in diet (50, 60, or 70%) for period of 42 d. ^b^ Price of feed 50% (USD 347.2/ton.), 60% (USD 352.76/ton.), and 70% (USD 358.31/ton.). ^c^ Includes metaphylactic antimicrobial treatment, vaccination, deworming, pour-on cypermethrin, and ear tag. ^d^ Price of CaPr,/kg: USD 4.38. ^e^ Calculated as (final body weight − initial body weight) × 3.62. The selling price by BW is USD 3.62. Feed cost/calf was calculated multiplying the cost of diet (USD/kg) × total feed consumed in the trial. * *p* < 0.05, ** *p* < 0.01, *** *p* < 0.001 without CaPr versus supplemented with CaPr.

## Data Availability

The information published in this study is available on request from the corresponding author.
